# Assessing diagnostic accuracy for asthma with home spirometry in primary care

**DOI:** 10.1038/s41533-025-00471-5

**Published:** 2025-12-26

**Authors:** Lynnea Myers, Martin Bellander, Henrik Ljungberg, Martine Isachsen, Marianne Eduards, Marie Lindman, Anna Carleborg, Maria Rosengren, Magnus Jansson, Hanna Sandelowsky, Kjell Larsson, Björn Nordlund

**Affiliations:** 1 Karolinska Institutet, Women and Children’s Health, Stockholm, Sweden; 2 Centre for Psychiatry Research, Department of Clinical Neuroscience, Karolinska Institutet & Stockholm Health Care Services, Stockholm, Sweden; 3 Karolinska University Hospital, Astrid Lindgren Children’s Hospital, Stockholm, Sweden; 4 Academic Primary Health Care Centre, Region Stockholm, Stockholm, Sweden; 5 Academic Primary Health Care Centre Jakobsberg, Region Stockholm, Stockholm, Sweden; 6 KTH Royal Institute of Technology, Division of Information Science and Engineering, Stockholm, Sweden; 7 Department of Neurobiology, Care Sciences and Society, Division of Family Medicine and Primary Care, Karolinska Institutet, Stockholm, SE Sweden; 8 Karolinska Institutet, Department of Environmental Medicine, Stockholm, Sweden

**Keywords:** Diseases, Health care, Medical research, Signs and symptoms

## Abstract

The aim was to evaluate the diagnostic accuracy of lung function measurements for asthma in primary care, including trial treatment. Undiagnosed patients seeking care for asthma-like symptoms were assessed at primary healthcare centers in Sweden. Participants underwent remote or in-clinic spirometry with bronchodilator responsiveness testing (BDR), remote diurnal variability testing of forced expiratory volume in 1 s (FEV_1_) and peak expiratory flow (PEF) over 2–4 weeks using a home spirometry system; and if necessary, three-months trial treatment with inhaled corticosteroids. Overall, 71/123 (58%) were diagnosed with asthma. When comparing patients by asthma diagnosis, sensitivity and specificity for documented diagnosis were 9% (95% CI 3–17) and 100% (93–100) for BDR; 61% (48–72) and 58% (43–71) for FEV₁; and 76% (64–85) and 69% (55–81) for PEF. Diurnal variability testing via home spirometry showed the strongest balance among sensitivity and specificity for asthma.

## Introduction

The global prevalence of asthma-like symptoms ranges from 1–33% in adults^1^ and approximately 10% in children^2^. Symptoms of asthma are recognised as cough, wheeze, shortness of breath, and chest tightness, which are more pronounced at night, in the morning, or during exertion. The Global Initiative for Asthma (GINA) 2024 defines asthma as a heterogeneous disease with chronic airway inflammation, variable respiratory symptoms, and fluctuating airflow limitations^3^.

Asthma is frequently under-diagnosed and under-treated in low- and middle-income countries^4^, while there is evidence of over-diagnosis and over-treatment in high-income countries^5^. There is a strong recommendation that the diagnosis is not based on symptoms alone^3,6,7^. Spirometry is a recommended diagnostic test per GINA guidelines^3^, typically through bronchodilator responsiveness testing (BDR) or by positive diurnal peak expiratory flow (PEF) variability. A European Respiratory Society task force has concluded that due to variable airway obstruction in asthma, BDR alone has a low sensitivity, but high specificity for asthma diagnosis^8^. Meanwhile, PEF variability testing is often underutilized in healthcare as it is considered time-consuming, challenging to manage, and lacks evidence^8–10^. The task force also notes that forced expiratory volume in one second (FEV_1_) and forced vital capacity (FVC) ratio correlate more closely with airway obstruction than PEF, for example^8^. GINA guidelines only currently recommend PEF measurements for variability^3^; however, a few studies have evaluated the potential value of FEV_1_ variability for diagnosis and the correlation between diurnal FEV_1_ and PEF variability^11–13^. With these clinical challenges and opportunities, it is advised to verify variable expiratory airflow limitation before treating asthma^3,14^.

Asthma diagnosis remains a global clinical challenge. There is currently no “gold standard” for asthma diagnosis in primary care settings. However, the goal of diagnosis is to determine the probability of asthma based on a combination of factors such as symptom assessment, objective lung function investigations, and patient response to treatment. Additionally, individuals with asthma experience fluctuating phases of symptoms, along with remission or relapse, which can lead to normal spirometry results in clinical settings^15^ and underdiagnosis of asthma^16^. Furthermore, conventional spirometry is resource-intensive, which limits its feasibility in everyday primary care. Home spirometry offers an alternative by enabling repeated, longitudinal lung function measurements and tracks symptoms in real-world settings. However, there is limited evidence the performance of home spirometry in relation to standard diagnostic pathways in primary care, particularly regarding diagnostic accuracy, patient adherence, and the ability to capture within-person variability that might be missed in typical one-time, clinic-based spirometry assessments. Using information gathered at home, along with history and assessments obtained in clinic, home spirometry may provide more comprehensive diagnostic decision-support^17^.

The regulatory agency-approved home spirometry system AsthmaTuner has been implemented in the Nordics and US healthcare system with more than 9000 active users, mostly in primary care. Evidence shows the self-management module improved treatment of uncontrolled asthma^18^, and the variability testing module reduced time for clinical decisions on occupational asthma diagnosis^19^. The aims of this study were to investigate the diagnostic accuracy of 1) dynamic spirometry with BDR in-person at the healthcare centre or remotely, 2) remote diurnal FEV_1_ and PEF variability testing using home spirometry for asthma, and finally, for the participants with an unclear diagnosis after the previous two investigations, 3) three-month trial treatment with inhaled corticosteroid (ICS). The manuscript follows the STARD^20^ checklist.

## Methods

### Study design

**Fig. 1:**
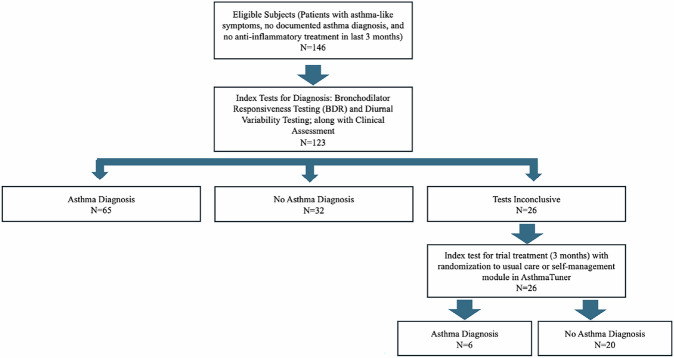
Study Flow of Participants in the ADVERT study.

The Asthma Diagnosis Verified by Lung Function (ADVERT) study was a real-world clinical study evaluating a pragmatic diagnostic protocol in the primary care setting (Fig. 1). Participants were consecutively recruited from seven primary healthcare centres (Liljeholmen VC, Ekerö VC, Jakobsberg VC, Ektorp VC, 5-Husläkare VC in Sollentuna, and Norrtälje Norra VC) from December 2020–December 2023 in Stockholm, Sweden. All participants in the study performed two to four weeks of diurnal FEV_1_ and PEF measurements remotely using a home spirometry system (AsthmaTuner) and BDR testing either in-clinic with stationary spirometry or remotely with AsthmaTuner. Spirometry measures performed remotely or in-clinic were quality assured in real-time. As a real-world study, doctors made independent decisions, reflecting routine clinical practice, on whether patients received an asthma diagnosis. These decisions were based on clinical assessment and/or diagnostic investigations, including data from both in-clinic diagnostic tests and remote testing via AsthmaTuner. If the diagnosis of asthma remained uncertain, participants received a three-month trial treatment with an ICS or ICS + LABA. These participants were randomly assigned (1:1) by nurses at each healthcare centre to either use spirometry with the self-management module in AsthmaTuner or follow a usual asthma treatment plan printed on paper. The study was approved by the Swedish Ethical Review Authority (ClinicalTrials.gov: NCT04652141) and participants provided written consents.

### Participants

Individuals seeking healthcare for asthma-like symptoms within the past month and at least six years of age were included. Individuals were excluded for the following reasons: intake of ICS or oral corticosteroids in the last three months, pregnancy, breast feeding, inability to perform spirometry, smoking history greater than ten pack-years, or severe medical condition (i.e., heart failure, aorta or cerebral aneurysm, history of myocardial infarction or stroke within the past three months).

### Patient and public involvement

The Swedish patient organisation for asthma and allergic diseases has been a key partner in developing the AsthmaTuner home spirometry system since 2015 and has voiced concerns about asthma misdiagnosis, particularly in primary care. Patients were involved from the point of recruitment, and clinical staff at each site contributed to the study’s design, recruitment, and conduct, with several also contributing to this manuscript.

### Electronic questionnaires

Participants completed questionnaires at baseline to collect demographic background factors, including smoking history, co-morbidities, and history of respiratory symptoms, treatment, and healthcare utilisation based on the European Community Respiratory Health Survey II (ECRHS II). A clinical report form was used for each participant to confirm study eligibility, ensure compliance with study procedures, document respiratory symptoms, and verify asthma diagnosis by code J.45 in the medical record.

### Home spirometry system

AsthmaTuner (MediTuner AB, Stockholm, Sweden) is a CE-marked cloud computing-based home spirometry system with a healthcare interface and patient mobile application (Android or iOS)^18^. The intended use of AsthmaTuner is to improve diagnosis and management of asthma. AsthmaTuner functions as an automated clinical decision support system by assisting with the diagnosis and management of asthma through three validated modules: remote diurnal variability testing based on FEV_1_ and PEF (Supplementary Figs. 1 and 2), dynamic spirometry testing including BDR (Supplementary Fig 3), and self-management support based on registered symptoms and measured FEV_1_ to provide immediate feedback on asthma control accompanied by an automated digital asthma action plan displaying the correct medication(s) and dose(s) prescribed by the user’s doctor (Supplementary Fig. 4)^18^. Using the AsthmaTuner web interface, healthcare providers can review spirometry with BDR, diurnal variability of FEV_1_ and PEF measurements, and patients’ symptom data in real time.

### BDR testing with dynamic spirometry

At the start of the study, all participants received dynamic spirometry coaching from a healthcare centre nurse. Forced vital capacity (FVC), forced expiratory volume in one second (FEV_1_) and forced mid-expiratory flow (FEF 25–75%) were measured at the study enrolment using spirometry at the healthcare centre or remotely with AsthmaTuner (Supplementary Fig. 3). Adult participants performed BDR testing using a recommended intake of four standard doses of salbutamol or terbutaline. Short- and long-acting beta2 agonists (SABA, LABA) were withdrawn eight and 24 h prior the testing, respectively. Predicted values of spirometry parameters were calculated based on the participant’s age, height, and sex at the start of the study using Global Lung Initiative (GLI) Standards from 2022 through the rspiro package (version 0.4). For participants using the AsthmaTuner device, the application’s software includes criteria from the American Thoracic Society/European Respiratory Society (ATS/ERS) for standardisation of lung function^21^, that must be fulfilled for the application to accept the maneuver on the spirometer.

### Diurnal variability testing

Participants were instructed to perform two to four weeks of at least twice daily FEV_1_ and PEF measurements at their convenience each day. The best of three FEV_1_ and three PEF readings were registered, to increase reliability. Only days with at least two measurements within the same day were selected for analyses. Daily variability was calculated by dividing the difference between the day’s highest and lowest FEV_1_ or PEF measurements by the mean of the same two FEV_1_ or PEF measurements (highest minus lowest diurnal divided by mean of highest and lowest measurements)^22^.

### Trial treatment

The trial treatment consisted of a single ICS inhaler or a combined ICS + LABA inhaler, whichever was ordered by the patient’s doctor. The dose was not standardized across patients. After three months of trial treatment, participants performed a new BDR testing. The doctor then re-evaluated the patient for asthma diagnosis.

### Main outcome

- Doctor’s diagnosis of asthma (yes/no) as recorded in the patient’s medical record after BDR testing with full spirometry and a minimum of 14 days of diurnal variability testing, and at least a three-month trial of treatment for inconclusive cases (Fig. 1). All participants had sought primary healthcare for asthma-like symptoms within the month prior to study entry, and diagnoses were confirmed (yes/no) at study end through individual review of medical records by primary care nurses at each participating centre.

### Definitions of spirometry outcomes

- BDR: Change greater than 12% and + 200 ml FEV_1_ from baseline to post-bronchodilator use^22^. We also applied the ATS/ERS guidelines^23^ with FEV_1_ (L) response greater than 10% (pre-bronchodilator minus post-bronchodilator FEV_1_ x 100 divided by predicted FEV_1_).
- Prebronchodilator FEV_1_/FVC for assessing airflow limitation using a fixed cut-off of 0.70^8^.
- Daily variability testing over two to four weeks: Number of days with positive diurnal variability greater than 10% for adults and 13% for children 6–11 years of age^22^.
- Positive trial treatment response: Change greater than 12% and + 200 ml FEV_1_ post-trial treatment^22^ (FEV_1_ before trial treatment subtracted from FEV_1_ after trial treatment x 100 divided by pre-trial treatment FEV_1_).

### Statistical methods

Differences between patients with and without doctor’s documented asthma diagnosis (see main outcome definition above) were analysed using chi-square test of independence for categorical variables, *t*-tests for non-skewed continuous variables, and Mann-Whitney U for skewed continuous variables. To be included in the sensitivity and specificity analyses for diagnostic accuracy, participants needed to complete BDR testing, at least one day of diurnal variability testing, and have a documented doctor’s diagnosis of asthma or no asthma diagnosis. The compliance for diurnal variability recordings was calculated as the percentage of readings performed out of readings possible during each individual’s evaluation period (typically ranging from 2–4 weeks). Sensitivity and specificity with 95% confidence intervals (CI) were calculated to assess diagnostic test performance of diurnal variability and BDR testing with the documented asthma diagnosis using MedCalc Software^24^. The Receiver Operating Characteristic (ROC) analyses were performed with the pROC package in R to determine optimal cut-off points and corresponding sensitivity and specificity using the closest.topleft method, which estimates the optimal cut-off value by minimizing the Euclidean distance between the ROC curve and the point (0,1). SPSS Version 29.0.1.0 and R version 4.4.1 were used for statistical analyses^25^.

A power analysis was performed to calculate the needed sample size. Aaron et al.^6^ reported that 14% of 613 individuals assessed for asthma had a positive BDR test. Assuming at least 28% of participants with asthma would have positive diurnal or weekly FEV_1_ or PEF variability of at least 10% or more, 146 participants needed to be included to get 80% power at a 5% significance level with adjustment for a 10% drop-out rate.

### Ethics approval

This study involves human participants and was approved by the Swedish Ethical Review Authority, 2020-04009, date: 2020-10-27. Participants provided informed consent prior to participation in the study. The study does not involve animal subjects.

## Results

### Participants

A total of 146 participants were recruited for the study. Of them, 23 (15.7%) participants were excluded from the diagnostic accuracy analysis of lung function measurements due to incomplete data (20 did not complete follow-up visit and/or had missing information on the primary outcome (asthma diagnosis), and three were immediately diagnosed with asthma and started asthma treatment (all completed diurnal variability testing, but only two did prebronchodilator BDR testing only before being diagnosed) (Fig. 1). This resulted in a final sample of 123 participants (67% female) with ages ranging from 7–66 years (mean 33 years). Of the 123 participants, 71 (58%) were diagnosed with asthma (six diagnosed after trial treatment), and 52 (42%) had no asthma diagnosis. Patients with no asthma diagnosis received some of the following diagnoses instead: cough, dyspnea, anxiety/pain, gastroesophageal reflux disease, sensory hyperactivity, viral infection (e.g. COVID-19), allergic disease, bronchitis, chest pain, or pollen allergy.

Overall, 24 (20%) participants reported a previous history of asthma. Participants reported a variety of asthma-like symptoms at the study start with the most frequent symptoms including wheezing, shortness of breath upon exertion, and nighttime coughing. Self-reported co-morbidities included diabetes, heart disease, migraine, neurological disease, celiac disease, and irritable bowel syndrome. Some baseline lung function measures (i.e., prebronchodilator FEV_1_ and FVC GLI percentages) did differ significantly between individuals with and without a diagnosis of asthma. Participant characteristics are presented in Table 1.

**Table 1:** Baseline Characteristics of the Study Population (n = 123).

Baseline Characteristics	Asthma Diagnosis (n = 71)	No Asthma Diagnosis (n = 52)†	p-value††
Age at inclusion, mean (SD, range)	32.4 (13.89; 11.19–66.72)	33.5 (17.24; 7.83–66.)	0.697
School children (<18 years)	13	12	
Female sex, n (%)	53 (74.6)	29 (55.8)	0.028
Ethnicity*			0.345
Caucasian, n (%)	32 (45)	37 (71)	
Asian, n (%)	2 (2.8)	1 (1.9)	
Other, n (%)	37 (52)	14 (27)	
Comorbidities, n (%)**	27 (38)	14 (27)	0.434
Ever had asthma, n (%)	18 (25)	6 (12)	0.165
Asthma attack in past 12 months, n (%)	10 (14)	1 (1.9)	0.338
Asthma medication, n (%)			0.316
Ever used	17 (24)	4 (7.7)	
Used in the past year	10 (14)	3 (5.8)	
Steroid asthma medication, n (%)			0.425
Ever used	10 (14)	2 (3.8)	
Used in the past year	3 (4.2)	2 (3.8)	
Ever had eczema, n (%)	32 (45)	16 (31)	0.221
Ever had allergic rhinitis, n (%)	47 (66)	29 (56)	0.719
Current smoker, n (%)	3 (4.2)	1 (1.9)	0.484
*Asthma-Like Symptoms (Last 12 months)*			
Wheeze, n (%)	40 (56)	22 (42)	0.227
Shortness of breath with wheeze, n (%)	34 (48)	17 (33)	0.615
Woken up with chest tightness, n (%)	24 (34)	14 (27)	0.653
Daytime shortness of breath, n (%)	12 (17)	11 (21)	0.538
Shortness of breath with exertion, n (%)	42 (59)	23 (44)	0.230
Nocturnal symptoms (i.e., cough), n (%)	32 (45)	27 (52)	0.605
Breathing problems, n (%)	52 (73)	32 (62)	0.352
Physical limitations, n (%)	13 (18)	3 (5.8)	0.126
*FEV*_*1*_			
Prebronchodilator, L, Mean (SD)	3.21 (0.67)	3.37 (0.92)	0.263
Prebronchodilator % Predicted Mean (SD)***	94.59 (11.24)	100.9 (13.53)	0.005
Postbronchodilator, L, Mean (SD)	3.37 (0.71)	3.46 (0.95)	0.585
Postbronchodilator % Predicted Mean (SD)***	99.54 (11.45)	103.58 (13.21)	0.073
*FVC*			
Prebronchodilator, L, Mean (SD)	4.11 (0.93)	4.26 (1.20)	0.434
Prebronchodilator % Predicted Mean (SD)***	101.40 (11.30)	106.24 (14.12)	0.037
Postbronchodilator, L, Mean (SD)	4.16 (0.98)	4.23 (1.20)	0.738
Postbronchodilator % Predicted Mean (SD)***	102.44 (11.65)	105.30 (14.13)	0.222
*FEV*_*1*_*/FVC*			
Prebronchodilator, L, Mean (SD)	0.79 (0.08)	0.80 (0.07)	0.468
Postbronchodilator FEV_1_/FVC, L, Mean (SD)	0.82 (0.07)	0.83 (0.07)	0.621

*SD* Standard Deviation, *FEV*_*1*_ Forced Expiratory Volume in 1 S, *L* litre, *FVC* Forced Vital Capacity.

*Ethnicity response categories included Caucasian, African, South East Asian, North East Asian, Other/Mixed.

**Co-morbidities included diabetes, heart disease, migraines, neurological disease, celiac, irritable bowel syndrome, other chronic gastrointestinal disease, autoimmune disease, cancer, or other chronic illness.

***Percent predicted based on Global Lung Function Initiative (GLI) values.

†No diagnosis of asthma means the participant did not receive a diagnosis of asthma from the doctor. Other diagnoses received include the following: cough, dyspnea, anxiety/pain, gastric reflux, sensory hyperactivity, viral infection (e.g., Covid-19, etc.), allergic disease, bronchitis, chest pain, pollen allergy.

††p-value for differences in those with and without a diagnosis of asthma calculated with Pearson Chi-Square for categorical variables, T-Test for non-skewed continuous variables, and Mann-Whitney U for skewed continuous variables.

### Diagnostic spirometry test

**Fig. 2:**
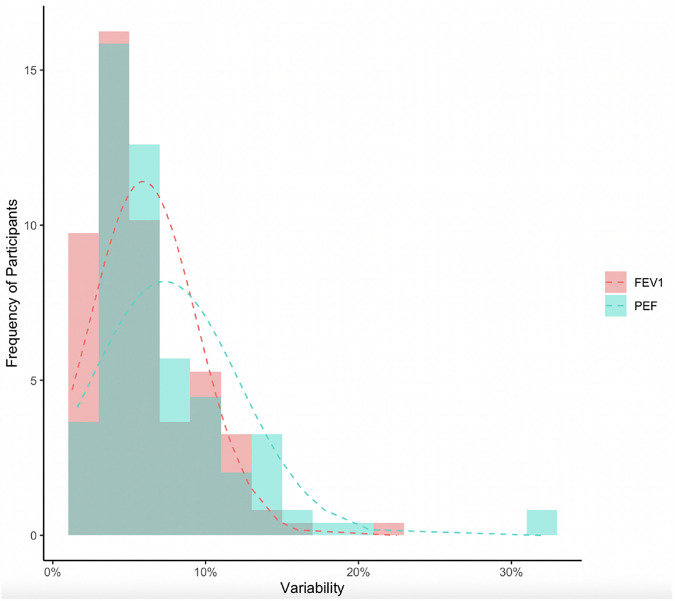
Histograms with normal distribution of averaged diurnal FEV_1_ and PEF variability during the period for participants.

BDR testing at the start of the study was positive in 6 of 123 (5%) participants per GINA guidelines and 7 of 123 (6%) participants per ATS/ERS 2022 guidelines. At least one day of positive diurnal variability for FEV_1_ and PEF was more common in diagnosed asthma cases versus those with no asthma diagnosis (FEV_1_ 76.1 vs. 55.8%, p = 0.018; PEF 91.5 vs, 63.5%, p < 0.001) (Table 2). Using an average of the diurnal variabilities obtained for each participant divided by number of days they performed the diurnal measurement, we found a Spearman’s correlation of r = 0.59 (p < 0.001) between FEV_1_ and PEF variabilities. The distributions of averaged diurnal FEV_1_ and PEF variabilities are presented in Fig. 2, indicating slightly wider distribution of positive diurnal variability when measured with PEF compared to FEV_1_.

**Table 2:** BDR and Variability Testing in Participants with All Diagnostic Information (n = 123).

Lung Function	Asthma Diagnosis, n = 71	No Asthma Diagnosis, n = 52	p-value*
**BDR Testing**			
Postbronchodilator FEV_1_ Change >12% & 200 ml, n (%)	6 (8.5)	0 (0)	0.032
ATS/ERS 2022 FEV_1_ Postbronchodilator Change >10%, n (%)	7 (9.9)	0 (0)	0.020
ATS/ERS 2022 FVC Postbronchodilator Change >10%, n (%)	5 (7.0)	0 (0)	0.051
**Variability**			
**Diurnal Variability**			
***FEV***_1_**	n (%)		
Any Day Diurnal Variability > 10%	54 (76.1)	29 (55.8)	0.018
2 + Days Diurnal Variability > 10%	43 (60.6)	22 (42.3)	0.045
3 + Days Diurnal Variability > 10%	22 (31.0)	15 (28.8)	0.798
4 + Days Diurnal Variability > 10%	17 (23.9)	9 (17.3)	0.373
FEV_1_ Highest Daily Diurnal Variability, median % (25^th^ and 75^th^ quartile; range)	13.8 (10.0, 20.4; 3.3–58.8)	12.2 (8.6, 16.0; 4.4–47.6)	0.039
PEF**	n (%)		
Any Day Diurnal Variability > 10%	65 (91.5)	33 (63.5)	<0.001
2 + Days Diurnal Variability > 10%	54 (76.1)	16 (30.8)	<0.001
3 + Days Diurnal Variability > 10%	42 (59.2)	11 (21.2)	<0.001
4 + Days Diurnal Variability > 10%	30 (42.3)	9 (17.3)	0.003
PEF Highest Daily Diurnal Variability, median % (25^th^ and 75^th^ quartile; range)	17.6 (13.2, 26.7; 3.5–67.6)	12.0 (9.0, 17.0; 4.5–40.6)	<0.001
**After Trial Treatment**			
Received Trial Treatment	6	20	0.612
AsthmaTuner Arm	4	11	
Traditional Arm	2	9	
Treatment Received During Trial Treatment			0.157
ICS	2	9	
ICS/LABA	4	11	
BDR			-
Postbronchodilator FEV_1_ Change >12% & 200 ml, n (%)	0	0	
ATS/ERS 2022 FEV_1_ Postbronchodilator Change >10%, n (%)	0	0	

*SD* Standard Deviation, *FEV*_*1*_ Forced Expiratory Volume in 1 S, *PEF* Peak Expiratory Flow, *GLI* Global Lung Function Initiative, *ATS/ERS* American Thoracic Society/European Respiratory Society.

*p-value for differences in those with and without a diagnosis of asthma calculated with Pearson Chi-Square for categorical variables, T-Test for non-skewed continuous variables, and Mann-Whitney U for skewed continuous variables. **Cut-off of 13% applied for children less 6–11 years.

Two days of positive diurnal variability tests over the evaluation period demonstrated the best combination of sensitivity and specificity, respectively, for asthma diagnosis: FEV_1_: 61%(48–72) and 58% (43–71) and PEF: 76% (64–85) and 69% (55–81) (Table 3). BDR testing using GINA guidelines had a sensitivity of 8.5% and specificity of 100% (Table 3).

**Table 3:** Analysis of the Sensitivity and Specificity of BDR Testing and Assessments of Diurnal Variability of FEV_1_ and PEF to Confirm Documented Asthma (n = 123).

	Asthma Diagnosis		
Test	Sensitivity (95% CI)	Specificity (95% CI)	AUC (p-value)**
**Variability Testing**			
***FEV***_***1***_			
Any Diurnal Variability (Binary > 10%)	76 (64–85)	44 (30–59)	
2 + Days Diurnal Variability > 10%	61 (48–72)	58 (43–71)	
3 + Days Diurnal Variability > 10%	31 (21–43)	71 (57–83)	
4 + Days Diurnal Variability > 10%	24 (15–36)	83 (70–92)	
***PEF***			
Any Diurnal Variability (Binary > 10%)	92 (83–97)	37 (24–51)	
2 + Days Diurnal Variability > 10%	76 (64–85)	69 (55–81)	
3 + Days Diurnal Variability > 10%	59 (47–71)	79 (65–89)	
4 + Days Diurnal Variability > 10%	42 (31–55)	83 (70–92)	
**Dynamic Spirometry**			
FEV_1_/FVC 0.7	82 (73–90)	9.6 (1.9–19)	0.48 (0.64)
FEV_1_/FVC* 0.8	56 (42–69)	55 (44–66)	
***Positive Response on BDR Testing***			
Postbronchodilator FEV_1_ Change >12% & 200 ml, n (%)	8.5 (3.2–17)	100 (93–100)	
2022 ATS/ERS Standards	9.9 (4.1–19)	100 (93–100)	
***Other***			
Positive on Both BDR Testing and Variability (Daily FEV_1_)	5.6 (1.6–14)	100 (93–100)	
Positive on Both BDR Testing and Variability (Daily PEF)	7.0 (2.3–16)	100 (93–100)	
	**Asthma Diagnosis**		
**Test**	**Sensitivity (95% CI)**	**Specificity (95% CI)**	AUC (p-value)**

*CI* Confidence Interval, *SD* Standard Deviation, *FEV*_*1*_ Forced Expiratory Volume in 1 S, *PEF* Peak Expiratory Flow, *ATS/ERS* American Thoracic Association/European Respiratory Society.

*Cutoff for optimal positive and negative predicted values in ADVERT.

**If applicable, for continuous variables.

The ROC analysis, assessing the ability of pre-bronchodilator FEV_1_/FVC to predict asthma diagnosis in real-world setting, showed that the optimal cut-off of 0.80 yielded 56% (42–69) sensitivity and 55%(44–66) specificity (Table 3). The fixed cut-off for FEV_1_/FVC 0.70 had a sensitivity of 9.6% (1.9–19) and specificity of 82% (73–90).

Trial treatment with ICS was administered to 26 participants with unclear diagnosis, resulting in six participants (23%) being diagnosed with asthma.

### Safety and compliance

No adverse events related to variability testing or self-management with the home spirometry system were reported. The mean percentage of compliance for diurnal variability measurements for all participants was 71% (range: 5.9–100%), with an average of 68% for participants diagnosed with asthma and 74% for participants with no asthma diagnosis (p = 0.08).

## Discussion

### Summary

In this real-world primary care study (ADVERT), most (79%) patients with asthma-like symptoms received a diagnostic decision based on BDR and diurnal variability tests conducted over a two- to four week period. Spirometry with BDR demonstrated the highest specificity, but had limited sensitivity for diagnosing asthma. The combination of at least 2 days or more of positive diurnal FEV_1_ or PEF variability during the evaluation period, together with clinical presentation of symptoms, provided the best balance of sensitivity and specificity for doctor-documented asthma diagnosis. The ROC analysis further suggested an optimal FEV_1_/FVC cut-off of 0.80, with a sensitivity of 56% (42–69) and a specificity of 55% (44–66). The findings of this study support the integration of home spirometry into routine diagnostic workflows in primary care as a complementary tool to enhance the accuracy of asthma diagnosis.

This study investigated the accuracy of home spirometry measurements for asthma, particularly for FEV₁, in addition to PEF, in diagnosing asthma. We found that two or more days of positive FEV1 or PEF diurnal variability measurements during the evaluation period could enhance diagnostic accuracy for asthma when considered alongside clinical symptom presentation. The patients evaluated in this study presented to primary healthcare centres with asthma-like symptoms, reflecting a higher pre-test probability of asthma. In such settings, lung function tests with higher sensitivity are often preferred to enable timely diagnosis and treatment, even if specificity is somewhat reduced.

### Comparison with existing literature

In contrast to GINA guidelines recommending at least two weeks of PEF monitoring^3^, our findings offer a novel perspective by suggesting that as few as two days of positive FEV_1_ or PEF diurnal variability measurements could be sufficient for diagnostic efficacy. This streamlined approach has the potential to expedite the diagnostic process with use of remote home spirometry system, allowing individuals experiencing asthma symptoms to receive more timely treatment and then to later undergo in-office procedures like BDR to get more detailed information on the severity of bronchial obstruction. Similarly, Korczynski et al.^26^ recently published a study where patients also performed twice-daily spirometry to identify airway obstruction. They too found that the use of remote spirometry demonstrated promising results to support asthma diagnosis. Remote spirometry may also reduce patient dropout rates, particularly among those reluctant to undergo prolonged diagnostic testing, while saving valuable time for healthcare providers who would otherwise need to administer and interpret extended testing protocols. Indeed, our previous study by Bjerg et al.^19^ found that the AsthmaTuner home spirometry system enabled accessible diagnostic testing for patients, helped support clinical decision-making for healthcare providers, and reduced the need for frequent clinical visits^19^. While we did also explore the value of using FEV_1_/FVC for asthma diagnosis, due to the limited sensitivity and specificity of the 0.80 cut-off observed, FEV₁/FVC should be combined with BDR or diurnal variability testing to improve asthma diagnostics, similar to the findings in the review by Louis et al.^8^.

In our study, a clinical decision regarding asthma diagnosis was reached in almost 80% of participants (53% for and 26% against) using a combination of BDR testing, diurnal variability, and clinical judgement. This is consistent with findings from a recent randomised clinical trial (RCT) by Aaron et al., which evaluated early diagnosis and treatment of asthma and chronic obstructive pulmonary disease (COPD) in individuals presenting with respiratory symptoms^27^. Similarly, Louis et al.^28^ found that combining clinical symptoms, particularly wheezing, with objective measures like spirometry and FeNO testing improved the prediction of an asthma diagnosis. It is worth noting that our study assessed baseline symptoms rather than the longitudinal symptom burden, which may explain why no differences in symptoms were observed between participants with and without an asthma diagnosis. Accurate and timely diagnosis and treatment for the millions of individuals with asthma worldwide can result in significant cost-savings. Indeed, the RCT by Aaron et al.^27^ pointed to significantly less healthcare use in participants assigned to undergo early diagnosis and treatment of asthma compared with usual care. Additionally, the potential value of more frequent data from home spirometry, including daily measurement of respiratory symptoms and lung function, may provide valuable data for the development of clinical decision support systems using artificial intelligence and prediction models^28^.

### Implications for research and/or practice

Digital tools like home spirometry enable collecting clinical data in a patient’s everyday life outside of the clinic setting. For example, home spirometry with AsthmaTuner demonstrated greater accuracy in diagnosing exercise-induced bronchial obstruction in athletes undergoing field-exercise challenge tests^29^. Furthermore, use of digital tools can contribute to more person-centred strategies implemented by the healthcare team, possibly resulting in fewer costly acute care visits or hospitalizations^27^ and better self-management skills in patients^18^. Although previous research shows inconsequent results for the reliability of home spirometry compared to in-clinic spirometry^30–34^, the use of healthcare professionals to coach patients in performing spirometry has consistently been shown to improve the validity of home spirometry^34–37^. The participants in our study received such coaching. They were able to successfully use the home spirometer coupled with the AsthmaTuner application to complete study procedures, and our study found stronger combinations of sensitivity and specificities for asthma diagnosis with diurnal measurements, supporting their validity for use. Acceptable compliance and feasibility of home spirometry systems used in primary care could help build relationships between patients and healthcare providers by making patients more involved in the diagnostic process^36,38,39^. As recommended in a recent review by Montemayor and Lechtzin^40^, further studies, particularly clinical trials, are needed to further determine the effect of home spirometry on clinical outcomes and which patients may benefit from its use. Regarding the potential for greater variability in home spirometry compared with in-office testing, it is worth noting that obtaining acceptable PEF or FEV₁ values is generally easier for patients than performing full spirometry, including acceptable FVC. The home spirometer used in this study incorporates integrated software algorithms that automatically validate each test according to ATS/ERS standards^21^. Specifically, for acceptability, the PEF must be reached within 30–160 ms, with no cough or Valsalva manoeuvre permitted. In line with these standards, at least three acceptable manoeuvres were required for each test, and the best independent PEF and FEV₁ values were used in the analysis.

### Limitations and strengths

Our study has limitations. First, it was performed in a single region in a country with a national health system; thereby potentially limiting the generalizability of findings to other locations. Since this was a real-world study, asthma diagnoses were made by doctors at each of the seven healthcare centres. Variability in doctor’s knowledge and experience in identifying and diagnosing asthma is a potential limitation, particularly since symptom profiles differed minimally between the asthma and non-asthma groups. If diagnostic guidelines such as GINA were not strictly followed, it is possible that some patients with asthma did not receive a diagnosis. While it would have been theoretically possible to employ a dedicated study doctor to standardise asthma diagnoses across all sites^27^ or to provide additional training to doctors at each site regarding asthma diagnosis, we opted for a real-world design with minimal intervention to enhance feasibility. The doctors in the study received no special training on asthma diagnosis; however, the nurses at each of the study sites received training on the use of the AsthmaTuner device and web-based care portal prior to the start of the study. This approach was chosen to provide valuable insights into how asthma is diagnosed in everyday clinical practice, which is essential for improving the translation of research findings into practical, real-world applications. As a real-world study, we did use the doctor’s diagnosis of asthma or other disease/disorder as the outcome. For any patients where the doctor was not certain of their diagnosis, they then were randomized to a three-month trial treatment, which was considered a sufficient period for the doctor to determine an asthma diagnosis according to guidelines. Although other diagnostic tests exist such as such as FeNO, blood eosinophils and bronchial challenge tests to potentially diagnose asthma, they were not used by the doctors in our study to support the diagnosis of asthma. These tests are generally performed in specialty (secondary) care in Sweden and not routinely provided in primary care. For FeNO specifically, it can be effective to rule-in asthma; however, it has limited sensitivity for asthma diagnosis, therefore, it cannot rule-out asthma^41–43^. Testing for blood eosinophils has poor diagnostic accuracy for asthma^43,44^. Bronchial challenge tests, such as methacholine and mannitol challenge tests, require specialized equipment and expertise, which makes these tests less feasible in primary care. They are also time-consuming and logistically challenging with multiple inhalation doses and repeated spirometry measurements, which makes these tests impractical for high-throughput primary care setting^41,42^. In addition, a negative bronchial challenge test does not rule out asthma due to the variable nature of airway hyperresponsiveness. It is important to note that because our study took place in the real-world setting, our sample size is therefore limited to patients we could recruit from clinical settings; however, we feel the strength of using the real-world setting, especially in a study around asthma diagnosis, was important. Finally, some of our exclusion criteria, namely the exclusion of smokers, limits generalizability of our findings to all primary care patients. We choose to exclude these patients due to the potential effects of COPD.

In conclusion, most undiagnosed patients with asthma-like symptoms in primary care were diagnosed based on presence of symptoms confirmed through BDR performed in-office or remotely, together with remote diurnal variability tests, without the need for trial treatment. Furthermore, two days of positive diurnal variability for FEV_1_ or PEF with a home spirometry system showed the best combination of sensitivity and specificity for diagnosing asthma. Future studies could confirm the diagnostic asthma accuracy of variability testing with home spirometry systems and verify the integration of such systems in various clinical settings and locations.

## Supplementary information

Supplementary Information

## Data Availability

Data from the study can be shared with additional ethical approval from the Swedish Ethical Review Authority. The full trial protocol available upon request.

## References

[1] Mortimer, K. et al. The burden of asthma, hay fever and eczema in adults in 17 countries: GAN Phase I study. *Eur. respiratory J.* **60**, 2102865 (2022). 10.1183/13993003.02865-2021 PMC9474894 35210319

[2] Asher, M. I. et al. Worldwide trends in the burden of asthma symptoms in school-aged children: global asthma network phase i cross-sectional study. *Lancet* **398**, 1569–1580 (2021). 34755626 10.1016/S0140-6736(21)01450-1 PMC8573635

[3] Global Initiative for Asthma. Global Strategy for Asthma Management and Prevention 2024 https://ginasthma.org.

[4] Diseases G. B. D., Injuries C Global burden of 369 diseases and injuries in 204 countries and territories, 1990-2019: a systematic analysis for the global burden of disease study 2019. *Lancet* **396**, 1204–1222 (2020).. 10.1016/S0140-6736(20)30925-9 PMC7567026 33069326

[5] Aaron, S. D., Boulet, L. P., Reddel, H. K. & Gershon, A. S. Underdiagnosis and overdiagnosis of asthma. *Am. J. respiratory Crit. care Med.* **198**, 1012–1020 (2018). 10.1164/rccm.201804-0682CI 29756989

[6] Aaron, S. D. et al. Reevaluation of diagnosis in adults with physician-diagnosed asthma. *JAMA* **317**, 269–279 (2017). 28114551 10.1001/jama.2016.19627

[7] Boulet, L. P. et al. A guide to the translation of the global initiative for asthma (GINA) strategy into improved care. *Eur. respiratory J.* **39**, 1220–1229 (2012). 10.1183/09031936.00184511 22282546

[8] Louis, R. et al. European respiratory society guidelines for the diagnosis of asthma in adults. *Eur. respiratory J.* **60**, 2101585 (2022). 10.1183/13993003.01585-2021 35169025

[9] Self, T. H., George, C. M., Wallace, J. L., Patterson, S. J. & Finch, C. K. Incorrect use of peak flow meters: are you observing your patients?. *J. Asthma* **51**, 566–572 (2014). 24720711 10.3109/02770903.2014.914218

[10] Csonka, L. L., Tikkakoski, A., Vuotari, L., Karjalainen, J. & Lehtimaki, L. Relation of changes in peak expiratory flow (PEF) and forced expiratory volume in 1 s (FEV(1)) during bronchoconstriction. *Clin. Physiol. Funct. Imaging* **44**, 447–453 (2024). 38923340 10.1111/cpf.12898

[11] Aggarwal, A. N., Gupta, D. & Jindal, S. K. The relationship between FEV1 and peak expiratory flow in patients with airways obstruction is poor. *Chest* **130**, 1454–1461 (2006). 17099024 10.1378/chest.130.5.1454

[12] Damaraju, V. et al. Agreement between forced expiratory volume in the first second (FEV(1)) and peak expiratory flow (PEF) in severe acute asthma. *Lung India* **39**, 484–487 (2022). 36629217 10.4103/lungindia.lungindia_223_22 PMC9623862

[13] Llewellin, P. et al. The relationship between FEV1 and PEF in the assessment of the severity of airways obstruction. *Respirology* **7**, 333–337 (2002). 12421241 10.1046/j.1440-1843.2002.00417.x

[14] Bateman, E. D. et al. As-needed budesonide-formoterol versus maintenance budesonide in mild asthma. *N. Engl. J. Med.* **378**, 1877–1887 (2018). 29768147 10.1056/NEJMoa1715275

[15] Aaron, S. D. et al. Overdiagnosis of asthma in obese and nonobese adults. *CMAJ* **179**, 1121–1131 (2008). 19015563 10.1503/cmaj.081332 PMC2582787

[16] Wang, R., Murray, C. S., Fowler, S. J., Simpson, A. & Durrington, H. J. Asthma diagnosis: into the fourth dimension. *Thorax* **76**, 624–631 (2021). 33504564 10.1136/thoraxjnl-2020-216421 PMC8223645

[17] van der Kamp, M. R., Hengeveld, V. S., Brusse-Keizer, M. G. J., Thio, B. J. & Tabak, M. eHealth technologies for monitoring pediatric asthma at home: scoping review. *J. Med. Internet Res.* **25**, e45896 (2023). 37477966 10.2196/45896 PMC10403763

[18] Ljungberg, H. et al. Clinical effect on uncontrolled asthma using a novel digital automated self-management solution: a physician-blinded randomised controlled crossover trial. *Eur. respiratory J.* **54**, 1900983 (2019). 10.1183/13993003.00983-2019 31481605

[19] Bjerg, A. et al. Shorter time to clinical decision in work-related asthma using a digital tool. *ERJ Open. Res.* **6**, 00259–02020 (2020). 32963995 10.1183/23120541.00259-2020 PMC7487349

[20] Cohen et al. STARD 2015 guidelines for reporting diagnostic accuracy studies: explanation and elaboration. *BMJ Open.* **6**, e012799 (2016). 28137831 10.1136/bmjopen-2016-012799 PMC5128957

[21] Miller, M. R. et al. Standardisation of spirometry. *Eur. respiratory J.* **26**, 319–338 (2005). 10.1183/09031936.05.00034805 16055882

[22] Global Initiative for Asthma. Global Strategy for Asthma Management and Prevention. 2023 Updated July 2023.

[23] Stanojevic, S. et al. ERS/ATS technical standard on interpretive strategies for routine lung function tests. *Eur. Respir. J.* **60**, 2101499 (2022). 34949706 10.1183/13993003.01499-2021

[24] Ltd. MS. Diagnostic test evaluation calculator Version 20.026:Available from: https://www.medcalc.org/calc/diagnostic_test.php.

[25] R Core Team. R: A Language and Environment for Statistical Computing. In: R Foundation for Statistical Computing, editor. Vienna, Austria: https://www.R-project.org/; 2023.

[26] Korczynski, P. et al. 30-day Spirometry Holter method design and prospective observational study. *Sci. Rep.* **14**, 26204 (2024). 39482397 10.1038/s41598-024-77803-x PMC11528101

[27] Aaron, S. D. et al. Early diagnosis and treatment of COPD and asthma - A randomized, controlled trial. *N. Engl. J. Med.* **390**, 2061–2073 (2024). 38767248 10.1056/NEJMoa2401389

[28] Louis, G. et al. Development and validation of a predictive model combining patient-reported outcome measures, spirometry and exhaled nitric oxide fraction for asthma diagnosis. *ERJ Open. Res.* **9**, 00451–02022 (2023). 36755965 10.1183/23120541.00451-2022 PMC9900444

[29] Reier-Nilsen, T. et al. Unsupervised field-based exercise challenge tests to support the detection of exercise-induced lower airway dysfunction in athletes. *BMJ Open. Sport. Exerc. Med.* **9**, e001680 (2023). 37520311 10.1136/bmjsem-2023-001680 PMC10373716

[30] Oppenheimer, J. et al. Clinic vs home spirometry for monitoring lung function in patients with asthma. *Chest* **164**, 1087–1096 (2023). 37385337 10.1016/j.chest.2023.06.029

[31] Wilson, C. L. et al. Home spirometry appears accurate and feasible for monitoring chronic respiratory disease. *ERJ Open. Res.* **10**, 00937–02023 (2024). 38770006 10.1183/23120541.00937-2023 PMC11103683

[32] Anand, R. et al. Unsupervised home spirometry versus supervised clinic spirometry for respiratory disease: a systematic methodology review and meta-analysis. *Eur. Respir. Rev.* **32**, 220248 (2023). 37673426 10.1183/16000617.0248-2022 PMC10481332

[33] Beaufils, F. et al. Adherence, reliability, and variability of home spirometry telemonitoring in cystic fibrosis. *Front. Pediatr.* **11**, 1111088 (2023). 36911035 10.3389/fped.2023.1111088 PMC9998040

[34] Wellmann, N. et al. Enhancing adult asthma management: a review on the utility of remote home spirometry and mobile applications. *J. Personalized Med.* **14**, 852 (2024). 10.3390/jpm14080852 PMC11355136 39202043

[35] Ramsey, R. R. et al. Feasibility and preliminary validity of mobile spirometry in pediatric asthma. *J. Allergy Clin. Immunol. Pract.* **9**, 3821–3823 (2021). 34153516 10.1016/j.jaip.2021.06.005 PMC8511135

[36] Schaffer, S., Strang, A., Shenoy, A., Selhorst, D. & Chidekel, A. Education and implementation of home spirometry in an adolescent cystic fibrosis population. *Respir. Med. Res.* **84**, 101040 (2023). 37734233 10.1016/j.resmer.2023.101040

[37] Koltowski, L. et al. Remotely supervised spirometry versus laboratory-based spirometry during the COVID-19 pandemic: a retrospective analysis. *Respir. Res.* **25**, 39 (2024). 38238745 10.1186/s12931-023-02586-0 PMC10797720

[38] Bindler, R. et al. Feasibility and acceptability of home monitoring with portable spirometry in young adults with asthma. *J. Asthma* **60**, 1474–1479 (2023). 36525469 10.1080/02770903.2022.2160345 PMC10191873

[39] M, R. osenfeld et al. Incorporating the perspectives of participants and research coordinators on home spirometry into clinical trial design: The example of the OUTREACH study. *J. Cyst. Fibros.* **23**, 739–743 (2024). 39079878 10.1016/j.jcf.2024.06.014

[40] Montemayor, K. & Lechtzin, N. Home Spirometry. *Clin. Chest Med.* **46**, 559–567 (2025). 40769599 10.1016/j.ccm.2025.04.014

[41] Simpson, A. J. et al. Asthma diagnosis: a comparison of established diagnostic guidelines in adults with respiratory symptoms. *EClinicalMedicine* **76**, 102813 (2024). 39296585 10.1016/j.eclinm.2024.102813 PMC11408836

[42] Kaya, T. T., Braunstahl, G. J. G., Veen, J., Kappen, J. H. J. & Valk, J. The Fractional exhaled Nitric Oxide (FeNO)- test as add-on test in the diagnostic work-up of asthma: a study protocol. *BMC Pulm. Med.* **24**, 178 (2024). 38622520 10.1186/s12890-024-02990-2 PMC11020987

[43] Nekoee, H. et al. Are type-2 biomarkers of any help in asthma diagnosis?. *ERJ Open. Res.* **6**, 00169–02020 (2020). 32714964 10.1183/23120541.00169-2020 PMC7369447

[44] Solomon, Y. et al. Peripheral blood eosinophilia in adult asthmatic patients and its association with the severity of asthma. *BMC Pulm. Med.* **23**, 96 (2023). 36949398 10.1186/s12890-023-02383-x PMC10031890

