# Supplementary material for: Assessing diagnostic accuracy for asthma with home spirometry in primary care
Source: NPJ Prim Care Respir Med. 2025 Dec 26;36:3. doi: 10.1038/s41533-025-00471-5 (PMC12774873; doi:10.1038/s41533-025-00471-5)
Supplement: Supplementary file 1 — Supplementary Information [file 41533_2025_471_MOESM1_ESM.docx]

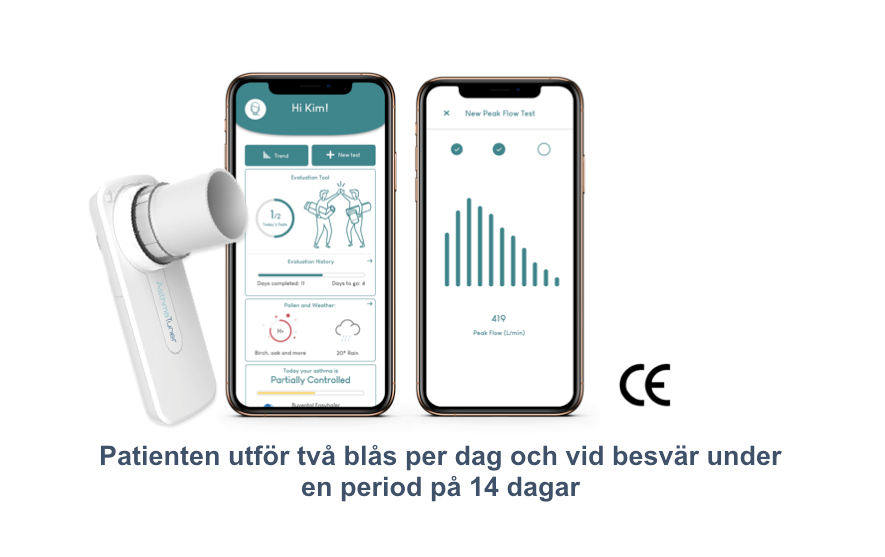


**Supplementary Figure 1.** AsthmaTuner diagnosis module enables daily measurement of PEF and FEV_1_.


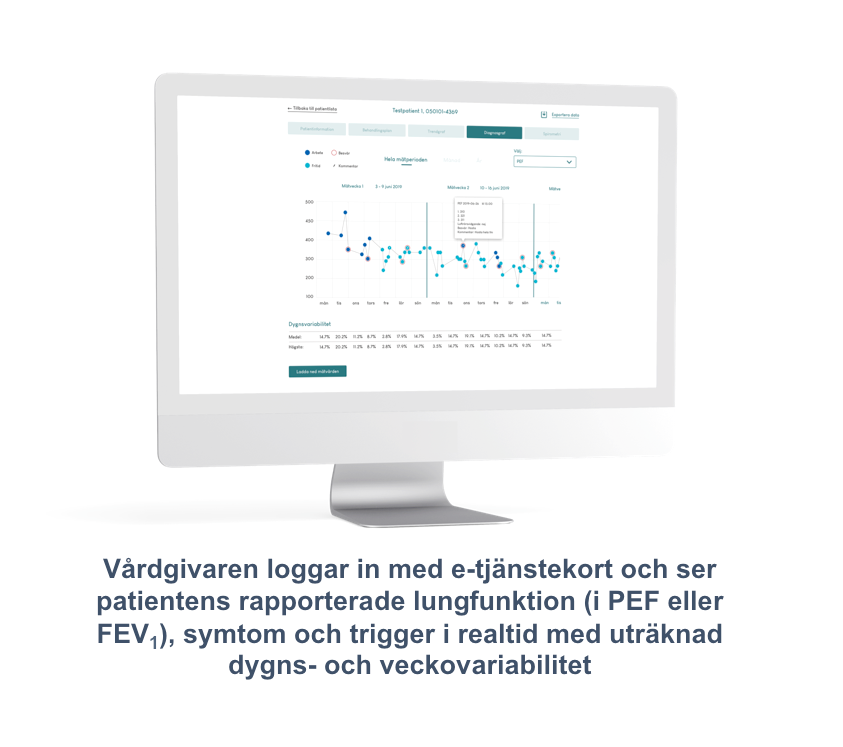


**Supplementary Figure 2.** The healthcare interface of AsthmaTuner provides diurnal and weekly calculations of variability in lung function in relation to symptoms and intake of asthma treatment.


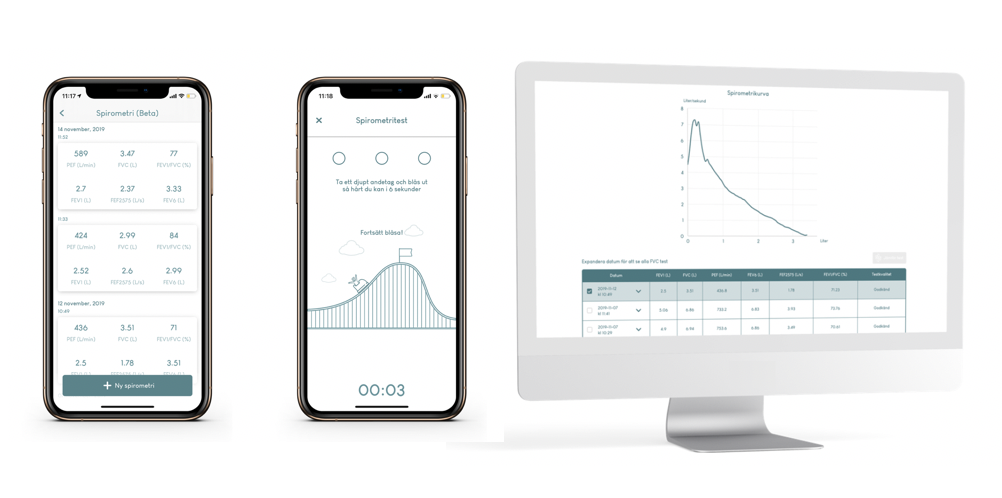


**Supplementary Figure 3.** Patients can perform spirometry with BDR using AsthmaTuner.


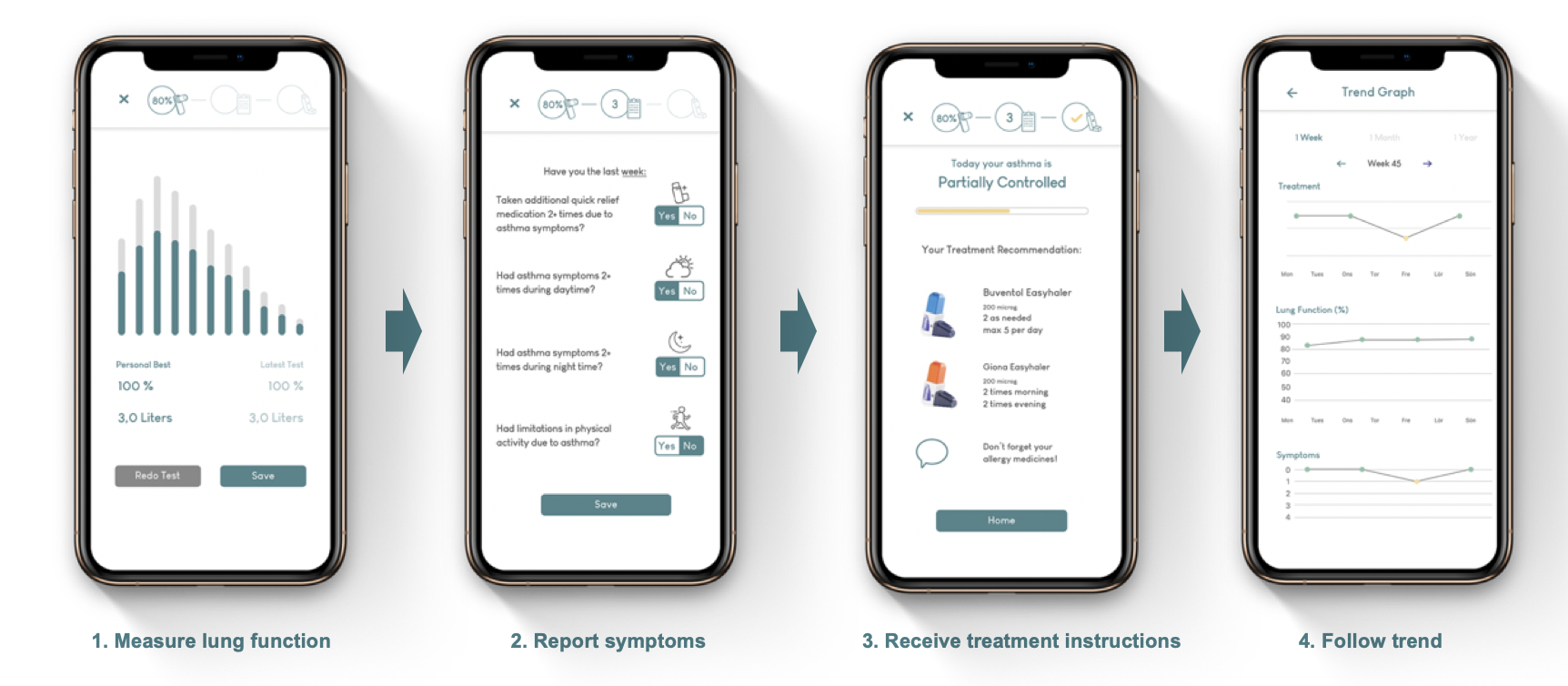


**Supplementary Figure 4.** The self-management app consists of a clinician-prescribed adjustable treatment plan and the use of an app and spirometer by participants to measure lung function, register perceived symptoms, and immediately receive feedback on current symptom control and treatment.
